# Clinical course, treatment and outcome of *Pneumocystis* pneumonia in immunocompromised adults: a retrospective analysis over 17 years

**DOI:** 10.1186/s13054-018-2221-8

**Published:** 2018-11-19

**Authors:** Julius J. Schmidt, Catherina Lueck, Stefan Ziesing, Matthias Stoll, Hermann Haller, Jens Gottlieb, Matthias Eder, Tobias Welte, Marius M. Hoeper, André Scherag, Sascha David

**Affiliations:** 1 Department of Nephrology and Hypertension, Hannover Medical School, Carl-Neuberg-Strasse 1, 30625 Hannover, Germany; 2 Department of Hematology, Hemostasis, Oncology and Stem Cell Transplantation, Hannover Medical School, Hannover, Germany; 3 Department of Microbiology, Hannover Medical School, Hannover, Germany; 4 Department of Clinical Immunology and Rheumatology, Hannover Medical School, Hannover, Germany; 5 Department of Pneumology, Hannover Medical School, and German Center for Lung Research (DZL), Hannover, Germany; 6 Institute of Medical Statistics, Computer and Data Sciences, Jena University Hospital, Jena, Germany; 7 Center for Sepsis Control and Care, Jena University Hospital, Jena, Germany

**Keywords:** Mortality, Transplantation, Lactate dehydrogenase, LDH, HIV

## Abstract

**Background:**

Despite modern intensive care with standardized strategies against acute respiratory distress syndrome (ARDS), *Pneumocystis* pneumonia (PcP) remains a life-threatening disease with a high mortality rate. Here, we analyzed a large mixed cohort of immunocompromised patients with PcP, with regard to clinical course and treatment, and aimed at identifying predictors of outcome.

**Methods:**

This was a single-center retrospective analysis in a tertiary care institution across 17 years. Diagnosis of PcP required typical clinical features and microbiological confirmation of *Pneumocystis jirovecii*. Epidemiological, clinical, laboratory and outcome data were collected from patient records.

**Results:**

A total of 52,364 specimens from 7504 patients were sent for microbiological assessment (3653 with clinical suspicion of *Pneumocystis* pneumonia). PcP was confirmed in 240 patients, about half of them HIV positive (52%). The remaining subjects were either solid organ transplant recipients (16.3%) or suffered from malignancy (15.8%) or autoimmune diseases (11.7%). Of note, 95% of patients with PcP were not receiving chemoprophylaxis. Overall in-hospital mortality was 25.4%, increasing to 58% if ICU admission was required. Multivariable regression identified lactate dehydrogenase (LDH) as predictor of in-hospital mortality (adjusted OR 1.17 (95% CI 1.09–1.27), *p* < 0.0001). Mortality in LDH quartiles increased from 8% to 49%, and a cutoff value of 495 U/L predicted mortality with sensitivity and specificity of 70%. With regard to treatment, 40% of patients received trimethoprim-sulfamethoxazole at doses that were lower than recommended, and these patients had a higher mortality risk (HR 1.80 (95% CI 1.10–3.44), *p* = 0.02).

**Conclusions:**

PcP remains a life-threatening disease among immunocompromised patients. About half of patients with PcP do not have HIV infection. Initial LDH values might serve as a stratifying tool to identify those patients at high risk of death among patients with HIV and without HIV infection.

**Electronic supplementary material:**

The online version of this article (10.1186/s13054-018-2221-8) contains supplementary material, which is available to authorized users.

## Background

*Pneumocystis* pneumonia (PcP) is a severe disease with high morbidity and mortality, which almost exclusively affects immunocompromised patients. PcP has long been known for its high prevalence among human immunodeficiency virus (HIV)-positive patients [1]. Since the implementation of combination antiretroviral therapies (cART) and chemoprophylaxis its incidence has been continuously decreasing [2]. Nowadays, most patients with HIV-associated PcP are treatment-naïve with very low CD4 cell counts; some of these patients do not know that they are HIV positive until they attend hospital [3]. On the other hand, PcP is also frequently diagnosed in non-HIV-positive patients as immunosuppressive regimens are being increasingly used in a wide range of patient populations. Consistently, the incidence of PcP in non-HIV-positive patients is increasing [2].

*Pneumocystis* infections have first been described in preterm infants following World War II [4] and in patients with malignancies in the late 1960s [5], more than a decade before the HIV epidemic emerged. Defects in cell-mediated immunity and use of glucocorticoids are among the strongest risk factors for the development of PcP [6, 7].

The epidemiology of PcP has been described in several retrospective studies [2, 8]. A study from the Mayo Clinic in 116 non-HIV-positive patients found that most frequent underlying diseases were hematological malignancies (30%), organ transplantation (25%), autoimmune disease (22%), solid tumors (13%) and other reasons [6]. Although, most of these reports were published more than a decade ago and might not reflect the current epidemiological situation, a report from France was published a few years ago [9]. Roux et al. reported overall mortality of 17.4%, which was significantly higher in non-HIV-positive patients (27%) than in HIV-positive patients (4%) [9]. A few parameters such as age, prior episode of PcP, low CD4 cell count, lactate dehydrogenase (LDH), and coinfections have been reported to predict unfavorable outcome in patients with HIV infection [10–12]. Reports on outcome predictors in non-HIV-positive patients are sparse.

Based on the high burden of PcP and the likelihood of unfavorable outcome particularly in non-HIV-positive patients, chemoprophylaxis with trimethoprim-sulfamethoxazole (TMP-SMX) is recommended in high-risk populations [13]. TMP-SMX is also the treatment of choice for PcP. Adjunctive corticosteroid therapy is recommended in HIV-positive patients with severe respiratory failure [14, 15]. However, while beneficial outcomes of higher dosage of corticosteroids in HIV-positive patients during PcP are reported [16], the outcome of using corticosteroids in non-HIV-positive patients is not clear [17].

We here report comprehensive epidemiological, clinical, laboratory, therapeutic and outcome data on 240 cases of PcP, including a high percentage of non-HIV-positive patients, in a tertiary care center over the last 17 years. Additionally, we aimed at identifying predictors of outcome.

## Methods

### Study design and population

We performed a retrospective, single-center cross-sectional analysis of all patients with a positive finding of *Pneumocystis jirovecii* on direct immunofluorescence testing or detection by Diff-Quick staining in the bronchial washing fluid or broncho-alveolar lavage (BAL) fluid, from January 2000 to June 2017. Our hospital is a university tertiary care center with approximately 1500 beds. Written informed consent was waived by the ethics committee due to the anonymized retrospective nature of the analysis.

All bronchial washing fluids or BAL samples from patients clinically suspected to have *P. jirovecii* pneumonia were evaluated within the study period. For every patient, clinical data on demographic characteristics, underlying disease, status of immune competence, treatment regimens of immunosuppression, PcP therapy regimen and mortality, were gathered in the study database. Date of diagnosis was defined as the date of microbiological confirmation. *P. jirovecii* direct immunofluorescence was performed using the Monofluo kit *P. carinii* (BioRad Laboratories, years 2000 to 2016) or the Detect IF *Pneumocystis carinii* kit (Axis-Shield Diagnostics Ltd., year 2017). Diff Quick staining was performed using the stain sets provided by Dade Behring or Siemens AG, respectively.

### Statistical analysis

Standard descriptive statistics were used to summarize the data (e.g. continuous: mean ± standard deviation/count: absolute and relative frequencies/time-to-event: Kaplan–Meier estimator). To identify predictors of in-hospital mortality or survival we applied logistic and Cox proportional hazards models. Consequently, both odds ratio (OR) and hazard ratio (HR) estimates were reported. To obtain multivariable adjusted estimates, all main effects with univariate *p* values less than or equal to 0.15 were investigated simultaneously. In addition, confidence intervals (CI) were calculated with coverage of 95%. Finally, we also created receiver operating characteristics (ROC) curves for in-hospital death, which were summarized by area under the curve (AUC) estimates. ROC analyses were based on an increasingly complex (leave one out) logistic regression model with in-hospital mortality as the predicted outcome and the following predictors, which were all entered linearly: LDH alone, LDH + age, LDH + age + body mass index (BMI), and for LDH + age + BMI + estimated glomerular filtration rate (eGFR). All reported *p* values are nominal and two-sided. In this explorative study, we applied a significance level of α = 0.05 (two-sided). All statistical analyses were done using GraphPad Prism 5.0 (La Jolla, CA, USA) or R 3.4.2.

## Results

### Description of study population and etiology of immunosuppression

**Fig. 1:**
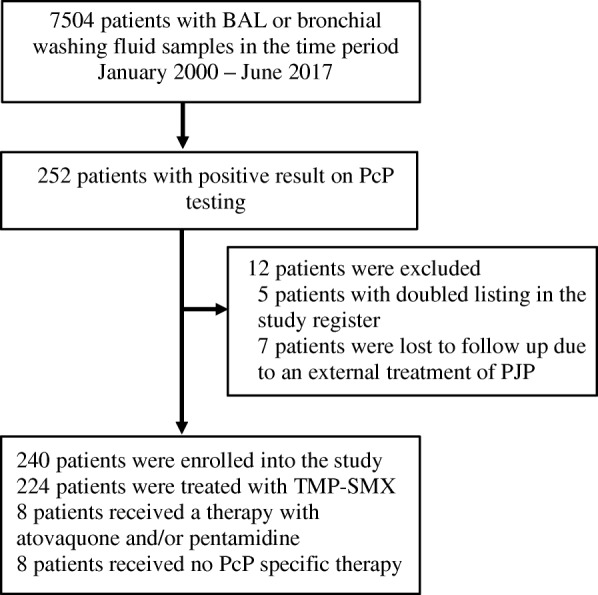
Flow chart. BAL, broncho-alveolar lavage; PcP *Pneumocystis* pneumonia, TMP-SMX, trimethoprim-sulfamethoxazole

During the observation period, 52,364 BAL or bronchial washing fluid samples were investigated. A clinical suspicion of *P. jirovecii* infection was raised in 3652 of these specimens of which 252 (i.e. 6.9%) were microbiologically confirmed by immunofluorescence as positive for PcP. There were ultimately 240 patients enrolled into the study (12 were excluded due to incomplete data sets) (Fig. 1). In the overall population, 67 patients were female (27.9%) and the average age was 45 ± 15 years. There were 125 patients (52%) with HIV infections; 39 (16.3%) had undergone solid organ transplant and 38 (15.8%) had undergone chemotherapy due to hematologic or oncologic malignancies: 28 patients (11.7%) received immunosuppressive therapy for rheumatoid autoimmune diseases (IS/RD) and 10 patients (4.2%) had other underlying diseases (miscellaneous (MISC)), e. g. common variable immune-deficiency (CVID) (Table 1). Five patients could be classified into multiple categories. After case reevaluation, patients were classified to the most recent active disease prior to the PcP event.

**Table 1:** Outcome and characteristics of the investigated study population of patients with PcP

	Overall 240 (100%)	HIV 125 (52%)	SOT 39 (16.3%)	ChTx 38 (15.8%)	IS/RD 28 (11.7%)	MISC 10 (4.2%)
Age (years)	44.8 ± 14.6	41 ± 10.6	49.5 ± 13.9^***^	49.7 ± 17.2^***^	51.2 ± 17.6^***^	37.3 ± 24.5
Women	67 (27.9%)	18 (14.4%)	11 (28.2%)	17 (44.7%)^♦♦♦^	15 (53.6%)^♦♦♦^	6 (60%)^♦♦^
ICU admission	100 (41.7%)	31 (24.8%)	19 (48.7%)^♦♦^	27 (71.1%)^♦♦♦^	16 (57.1%)^♦♦^	7 (70%)^♦♦^
Mechanical ventilation	88 (36.7%)	26 (22.4%)	17 (43.6%)^♦^	21 (55.3%)^♦♦♦^	16 (57.1%)^♦♦♦^	6 (60%)^♦^
CRRT	39 (16.3%)	4 (3.2%)	19 (48.7%)^♦♦♦^	6 (15.8%)^♦^	7 (25%)^♦♦♦^	3 (30%)^♦♦^
ECMO/ECLA treatment	11 (4.6%)	5 (4%)	3 (7.7%)	2 (5.3%)	1 (3.6%)	0 (0%)
in-hospital mortality (%)	61 (25.4%)	16 (12.8%)	15 (38.5%)^♦♦^	17 (44.7%)^♦♦♦^	10 (35.7%)^♦♦^	3 (30%)
TMP-SMX prophylaxis (%)	12 (5%)	6 (4.8%)	1 (2.6%)	5 (13.2%)	0 (0%)	0 (0%)
initial LDH (U/L)	502.1 ± 281.6	482.2 ± 247.2	482.5 ± 374.0	498.0 ± 236.4	627.3 ± 347.2 ^*^	493.5 ± 160.5

*Abbreviations*: *PcP* Pneumocystis pneumonia, *ICU* intensive care unit, *CRRT* continuous renal replacement therapy, *ECMO* extracorporeal membrane oxygenation, *ECLA* extracorporeal lung assist, *TMP-SMX* trimethoprim-sulfamethoxazole, *HIV* human immunodeficiency virus, *SOT* solid organ transplantation, *ChTx* chemotherapy, *IS/RD* immunosuppression/rheumatic diseases. *MISC* miscellaneous

Difference compared to HIV-positive patients (Fisher’s exact test): ^♦^*p* < 0.05, ^♦♦^*p* < 0.01, ^♦♦♦^*p* < 0.001

Difference compared to HIV-positive patients (Mann–Whitney U test): ^*^p < 0.05, ^**^p < 0.01, ^***^p < 0.001

### Clinical course of *Pneumocystis* pneumonia

**Fig. 2:**
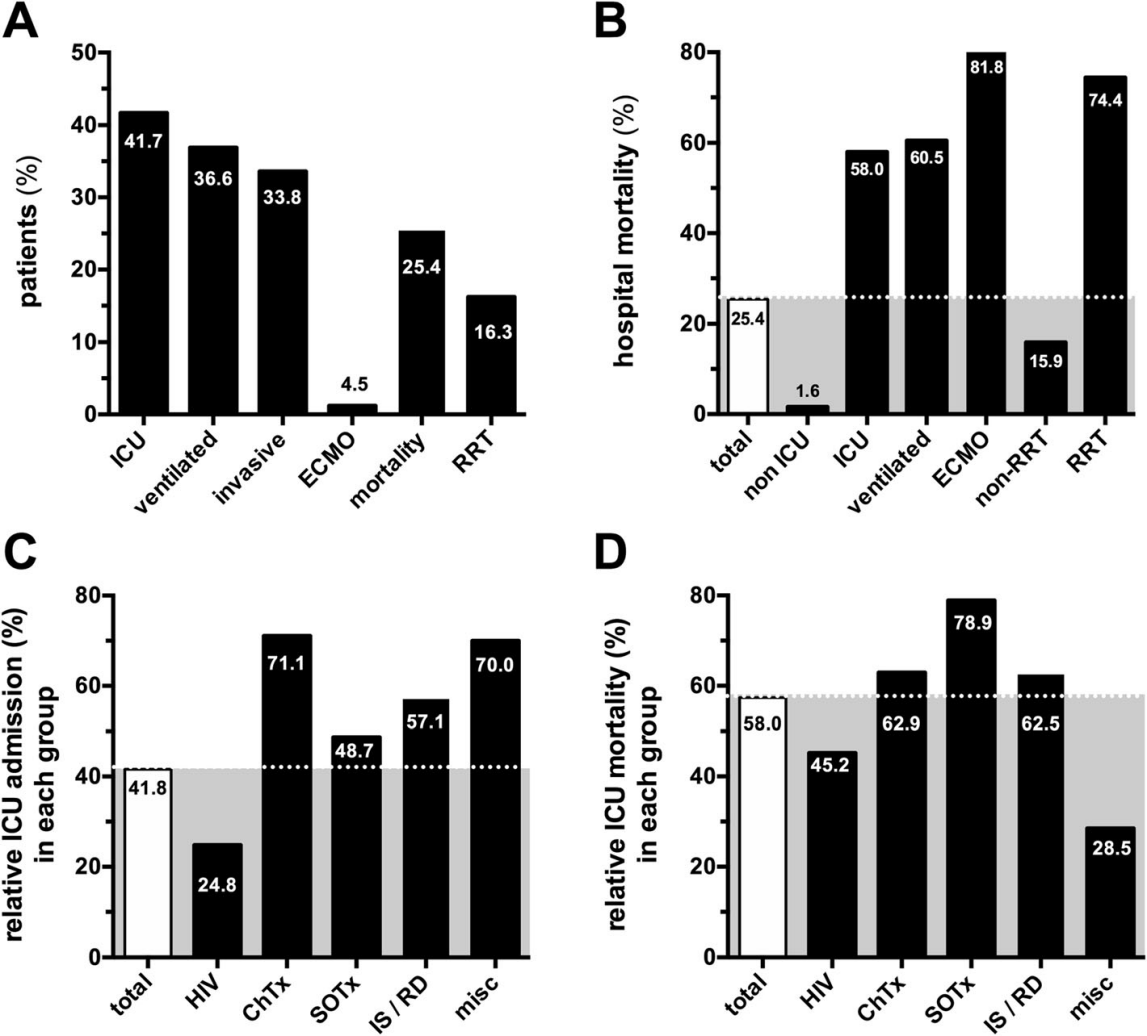
Etiology and clinical course of *Pneumocystis* pneumonia (PcP). **a** Percentage of patients with respect to clinical course of PcP including organ replacement therapies (ICU, intensive care unit; ECMO, extracorporeal membrane oxygenation; RRT, renal replacement therapies) and outcomes. **b** Hospital mortality in different clinical settings (gray area highlights overall mortality). **c** Percentage of ICU admission and **d** percentage of ICU mortality with immunosuppression with different etiologies. The gray area shows the total percentage independent from the etiology. SOT, solid organ transplant; ChTx, chemotherapy; IS/RD, immunosuppression/rheumatic disease

The average cumulative incidence of PcP in our institution was 13 ± 5 cases per year, with a peak in the years 2005–2010 (Additional file 1: Figure S1A). Of the 240 patients with PcP, 41.7% were admitted to the intensive care unit (ICU), with 36.6% in need of mechanical ventilation, 16.3% in need of renal replacement therapy (RRT), and 4.5% in need of extracorporeal membrane oxygenation (ECMO) support (Fig. 2a). The overall in-hospital mortality was 25.4%. The mortality was 58% in patients requiring ICU treatment, 74.4% in patients requiring RRT and 81.8% in patients requiring ECMO support, and only 1.6% of patients died on regular (non-ICU) wards (Fig. 2b). Mortality during the observation time from 2000 to 2017 slightly fluctuated but did not trend toward an improvement in more recent years (Additional file 1: Figure S1B).

The underlying disease was associated with outcome (Table 1). The lowest mortality was observed in HIV-infected patients (12.8%); the respective mortality rates in the non-HIV group were 38.4% in solid organ transplant recipients, 30.0% in patients with rheumatic diseases and 44.7% in patients with hematologic-oncologic diseases, respectively (Additional file 1: Figure S1C). Figure 2c, d summarizes ICU admission and ICU mortality with regard to the etiology of the patients’ immunosuppressive disease. Of note, only 12 patients (5%) were receiving chemoprophylaxis with TMP-SMX at the time when PcP was diagnosed (Table 1).

### Predictors of in-hospital mortality

Univariate regression analysis revealed that age, BMI, GFR and initial LDH were associated with death from PcP. Both logistic and cox multivariable regression analyses identified LDH as a predictor of unfavorable outcome in PcP (Table 2, adjusted OR 1.17 (95% CI 1.09–1.27) per 50 U/L, *p* < 0.0001, adjusted HR 1.07 (95% CI 1.04–1.10) per 50 U/L, *p* < 0.0001).

**Table 2:** Regression model results for in-hospital mortality and overall survival in 240 patients with PcP underlining the role of LDH

Patient characteristic (potential predictors)^a^	Logistic regression models for in-hospital mortality						Cox regression models for overall survival					
	Univariate models			Multivariable model^b^			Univariate models			Multivariable model^c^		
	Odds ratio	95% CI	*p* value	Odds ratio	95% CI	*p* value	Hazard ratio	95% CI	*p* value	Hazard ratio	95% CI	p value
LDH (50 U/L)	1.16	(1.10–1.24)	1.5 × 10^−6^	1.17	(1.09–1.27)	3.7 × 10^−5^	1.08	(1.06–1.11)	1.2 × 10^−9^	1.07	(1.04–1.10)	3.2 × 10^− 5^
Age (5 years)	1.28	(1.15–1.44)	1.9 × 10^− 5^	1.66	(0.82–3.87)	0.205	1.14	(1.05–1.24)	0.001	1.33	(0.76–2.32)	0.311
Sex (ref. male)	1.23	(0.65–2.31)	0.515				1.30	(0.81–2.09)	0.285			
BMI (kg/m^2^)	1.14	(1.06–1.23)	1.5 × 10^−4^	0.66	(0.33–1.33)	0.229	1.11	(1.05–1.17)	1.7 × 10^−4^	0.65	(0.40–1.06)	0.082
Admission GFR (10 mL/min/1.73 m^2^)	0.90	(0.84–0.97)	0.003	0.80	(0.60–1.07)	0.121	0.96	(0.91–1.01)	0.084	1.01	(0.80–1.28)	0.914
Prednisone equivalent dose (50 mg/day)	1.52	(1.13–2.13)	0.010	1.32	(0.96–1.85)	0.083	1.08	(0.95–1.23)	0.231	1.04	(0.89–1.21)	0.626
TMP (10 kg/day)	0.70	(0.42–1.16)	0.166				0.87	(0.60–1.27)	0.469			

*Abbreviations: PcP* Pneumocystis pneumonia, *LDH* lactate dehydrogenase, *BMI* body mass index, *TMP* trimethoprim-sulfamethoxazole, *CI* confidence interval

^a^Unit for which effect was estimated or reference (ref.) in parenthesis

^b^Multiple logistic regression including all predictors that met *p* ≤ 0.15 in one of the univariate analyses for both in-hospital mortality and overall survival; note that we also included quadratic terms for the continuous predictors (results not reported)

^c^Multiple Cox regression including all predictors that met *p* ≤ 0.15 in one of the univariate analyses for both in-hospital mortality and overall survival; note that we also included quadratic terms for the continuous predictors (results not reported)

**Fig. 3:**
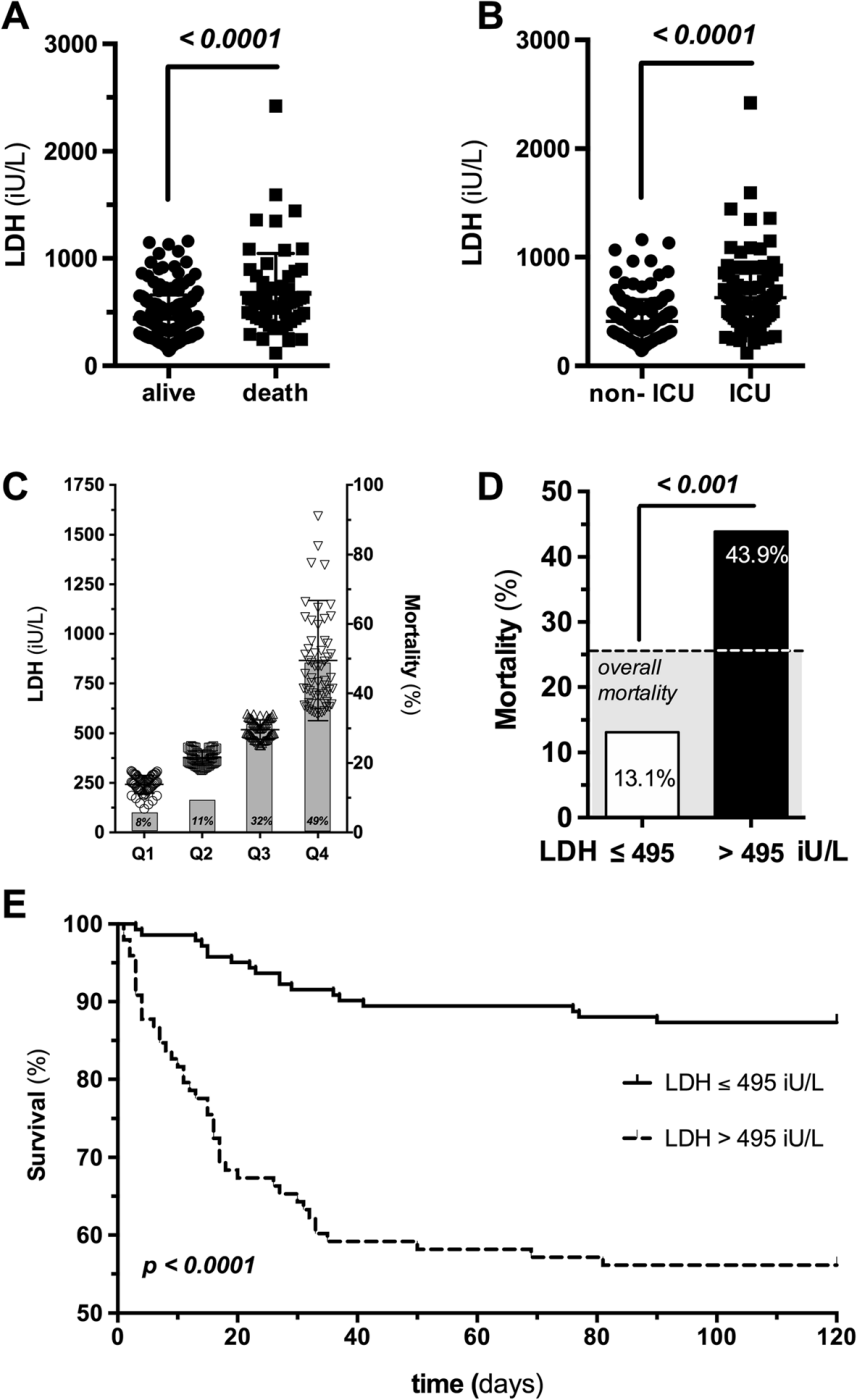
Influence of initial lactate dehydrogenase (LDH) as an outcome predictor. The scatter plot shows initial LDH levels at admission in later survivors (alive) and non-survivors (dead) (443 ± 214 vs. 673 ± 373 iU/L, *p* < 0.0001) (**a**) and in non-ICU versus ICU patients (411 ± 199 vs. 627 ± 328 iU/L, *p* < 0.0001) (**b**). **c** Bar graph showing overall mortality (right y-axis) stratified for initial LDH quartiles (left y-axis) (Q1, < 310; Q2, 311–436; Q3, 437–599; Q4, > 600 iU/L). **d** Mortality in all patients with *Pneumocysti*s pneumonia with LDH levels ≤ and > 496 iU/L (cutoff value was derived from ROC curve of all patients, with sensitivity and specificity of 70%.) The gray area highlights the overall mortality of 24.8% without LDH risk stratification**. e** Kaplan–Meier analysis of survival in patients stratified for LDH ≤ and > 495 iU/L during 120 days (*p*_log-rank test_ < 0.0001)

The initial LDH levels were significantly higher in later non-survivors (443 ± 214 vs. 673 ± 373 iU/L, p < 0.0001, Fig. 3a) and in patients that required admission to the ICU (411 ± 199 vs. 627 ± 328 iU/L, *p* < 0.0001, Fig. 3b). Stratification of patients into LDH quartiles showed increasing (in-hospital) mortality rates among those. Mortality ranged between 8% in the lowest and 49% in the highest LDH quartile (Fig. 3c). A ROC curve for in-hospital death had an estimated AUC of 0.724 (95% CI 0.65–0.80) (*p* < 0.0001 for LDH alone, Additional file 1: Figure S2). Potential alternative cutoff values and their impact on usually reported statistics are summarized in Additional file 2: Table S1 for descriptive purposes. We used a cutoff of 496 iU/L for further analysis. Patients with initial LDH below 496 iU/L had lower mortality than the overall population (13.1 vs. 25.4%) and lower mortality than those with initial LDH > 496 iU/L (13.1 vs. 43.9%, *p* < 0.001, Fig. 3d, e).

The addition of other clinical quantitative variables to LDH in the AUC model such as age, BMI and eGFR did not improve the predictive value of LDH alone (Additional file 1: Figure S2, Additional file 2: Table S2) in the overall cohort. However, when ICU patients were analyzed separately, we observed that the addition of age as a variable increased the predictive performance of LDH alone from an AUC of 0.61 (95% CI 0.49–0.72) to an AUC of 0.71 (95% CI 0.60–0.81) (*p* = 0.024). Of note, we did not detect any etiology subgroup that showed better performance in the ROC analysis (Additional file 1: Figure S3), nor did we detect temporal trends when applying the LDH prediction models in three strata (2000–2005, 2005–2010, 2010–2017) (Additional file 1: Figure S4).

### Treatment patterns and outcome

There were 224/240 patients (93.3%) initially treated with the gold standard, TMP-SMX. The remaining 16 patients had alternative regimens with chemotherapeutics from the second line, mostly due to side effects and intolerance of TMP/SMX (e.g. atovaquone and/or pentamidine, Fig. 1). Among the 224 patients treated with TMP-SMX, 159 (71%) were dosed in the recommended range above 15 mg/kg bodyweight (BW) per day, while 65 patients (29%) received a dose lower than 15 mg/kg BW (Additional file 1: Figure S5A). Of note, these patients had a higher mortality risk (HR 1.80 (95% CI 1.10–3.44), *p* = 0.02). Solid organ transplant (SOT) recipients and patients with rheumatoid diseases were more likely to receive lower dosages (Additional file 1: Figure S5B). On the other side, we observed that under-dosed patients also had significantly lower eGFR (48.9 ± 43 ml/min vs. 104 ± 32 ml/min, *p* < 0.0001). Interestingly, the absolute dose of TMP-SMX was not different in survivors (16.9 ± 5.5 mg/kg) and non-survivors (15.6 ± 7.1 mg/kg, *p* = 0.93). Still, Kaplan–Meier survival curves revealed a significant difference when patients were grouped into those treated with < 15 mg/kg and those treated with ≥ 15 mg/kg TMP-SMX (Additional file 1: Figure S5C, *p*_log-rang test_ = 0.02). However, TMP-SMX dose did not independently predict outcome in the multivariable regression analyses.

## Discussion

Here we present the single-center experience with regard to epidemiology, treatment and outcome of PcP in a tertiary care center over a period of 17 years. A substantial group of our PcP cases (about 50%) were not related to HIV infections and these patients had a worse clinical course with higher ICU admission and mortality rates than patients with HIV-associated PcP. Three major non-HIV-positive groups were identified: (1) solid organ transplant recipients, (2) patients with malignancies and (3) patients with rheumatic diseases. PcP occurred almost exclusively in patients who did not receive chemoprophylaxis with TMP-SMX. Overall, about one quarter of all patients suffering from PcP did not survive the disease. In those who required intensive care (approximately 40% of all patients), the in-hospital mortality increased up to 58%. Based on the - in principle - reversible nature of PcP, almost no patients were denied intensive care in the event of acute respiratory failure, as the in-hospital mortality rate in non-ICU patients was 1.6%. Moreover, we found that the extent of initial LDH elevation was a predictor of in-hospital mortality, and that in univariate comparisons, in-hospital mortality was higher in patients whose TMP-SMX dose was below 15 mg/kg BW.

Non-HIV-positive populations at risk of PcP have been described before. The distribution across these risk groups varies widely (SOT 3–31%, hematologic diseases 26–39%, rheumatic diseases 11–25%), which is likely due to the clinical emphasis in predominantly monocentric studies [9, 18–20]. Several centers have reported a decrease in the percentage of HIV-infected patients among all cases of PcP in the era of cART [19, 21]. We did not confirm this decrease, nor did Fillatre et al. [18].

Our data showed that the clinical course and outcome of PcP differed between HIV-infected and non-HIV-infected patients. In general, HIV-infected patients require fewer ICU admissions and have better overall survival than non-HIV-infected patients, which is in line with previous reports [9, 22]. The PcP mortality rate in HIV-positive patients was in the range of 1–15% [9, 18, 19] compared to 30–40% in HIV-negative patients [23–25]. The better outcome of HIV-positive patients might be the consequence of (1) lower age (− 3.8 years in our cohort), (2) fewer co-morbidities, (3) the reversible nature of the immune defect upon anti-viral treatment strategies and hypothetically (4) greater awareness of PcP in HIV-positive individuals, where it is the most common AIDS-defining disease.

In mixed cohorts, ICU admission and mechanical ventilation occurred in 22–40% and 13–22% of patients, respectively [9, 18, 22]. Both events are frequently reported as negative predictors of survival. Nevertheless, newer data on ICU mortality in patients with PcP are scarce. Only one recent study reported relatively high ICU mortality in HIV-negative patients of 53% compared to 15% in HIV-positive patients [18]. Mortality in patients with invasive mechanical ventilation was 60% and 28%, respectively, in these two groups [22]. Compared to other reports [21, 26], we had a relatively high ICU admission rate of 25% in HIV-positive patients, which can in part be explained by the selective transfer of more patients with more progressive disease to our university hospital as a tertiary center.

The literature is inconclusive on outcome predictors, but on multivariable analysis, a large prospective study from France demonstrated a significant survival benefit in younger patients [9]. Although observed in HIV-positive patients before [27], here we report for the first time that in a mixed population of HIV-positive and non-HIV-positive patients the initial levels of serum LDH at hospital admission are not only useful in the diagnostic evaluation [28] but may also be an independent predictor of survival. This might potentially help the clinician in early identification of the sickest patients at high risk of unfavorable outcome. With regard to the specificity/sensitivity, the ideal LDH cutoff value to predict outcome has yet to be defined.

With regard to the actual treatment of PcP we found that a reduction in the recommended TMP-SMX dose below 15 mg/kg BW (for whatever reason) might be associated with higher mortality (13.1 vs. 55.8%). Given that this observation was not confirmed in the multivariable analysis, relevant confounders must be considered.

Our study has several limitations. This was a retrospective observational study based on the medical records of patients with PcP from a single institution. Consequently, causal claims cannot be made. The observation that under-dosing with TMP-SMX is associated with higher mortality might be confounded by a higher percentage of patients with renal impairment. More robust evidence from well-designed studies is needed to make firm conclusions or even to make implications about changes in standard PcP management.

## Conclusions

In summary, PcP is a rare but potentially fatal disease in immunocompromised patients with diseases of different etiology. About 50% of cases were non-HIV-associated. Initial LDH levels - if validated by others - might be a useful predictor of in-hospital mortality, and TMP-SMX treatment doses in patients at high risk of death (e.g. ICU admission + LDH > 495 U/L) should probably not be reduced below 15 mg/kg BW.

## Additional files

Additional file 1: **Figure S1.** Cumulative incidence of (A**)** and in-hospital mortality in (B) *Pneumocystis* pneumonia (PcP) at Hannover Medical School from 2000 to 2017. **Figure S2.** Receiver operating characteristic (ROC) curves for in-hospital mortality applied to the total sample. **Figure S3.** ROC curves for in-hospital mortality applied to subgroups regarding underlying etiology of immunosuppression and for the ICU cohort. **Figure S4.** ROC curves for in-hospital mortality with the LDH prediction model applied in three strata (years 2000–2005, 2005–2010, 2010–2017). **Figure S5.** Association between trimethoprim-sulfamethoxazole (TMP-SMX) dose and mortality (DOCX 21 kb)

Additional file 2: **Table S1.** Suggested, alternative data-derived LDH cutoff values and their impact on usually reported statistics (with 95% confidence interval in parenthesis) related to the prediction of in-hospital mortality in patients with PcP. **Table S2.** Estimated AUCs (with 95% confidence interval) of the LDH-related logistic regression models for the prediction of in-hospital mortality in patients with PcP. **Table S3.** Additional descriptive information (with 95% confidence interval in parenthesis) on patient characteristics and their potential value as predictors of in-hospital mortality in patients with PcP. **Table S4.** Estimated AUCs (with 95% confidence interval) of the LDH-related logistic regression models for the prediction of in-hospital mortality in patients with PcP. (DOCX 10367 kb)

## Abbreviations

AUC: Area under the curve
BAL: Broncho-alveolar lavage
BMI: Body mass index
BW: Bodyweight
cART: Combination antiretroviral therapies
CI: Confidence interval
CRRT: Continuous renal replacement therapy
CVID: Common variable immune-deficiency
ECMO: Extracorporeal membrane oxygenation
eGFR: Estimated glomerular filtration rate
HIV: Human immunodeficiency virus
HR: Hazard ratio
IS: Immunosuppressive
LDH: Lactate dehydrogenase
OR: Odds ratio
PcP: *Pneumocystis* pneumonia
RD: Rheumatic disease
ROC: Receiver operating characteristic curve
SOT: Solid organ transplantation
TMP-SMX: Trimethoprim-sulfamethoxazole
