# Supplementary material for: Clinical course, treatment and outcome of Pneumocystis pneumonia in immunocompromised adults: a retrospective analysis over 17 years
Source: Crit Care. 2018 Nov 19;22:307. doi: 10.1186/s13054-018-2221-8 (PMC6245758; doi:10.1186/s13054-018-2221-8)
Supplement: Supplementary file 1 — Figure S1. Cumulative incidence of (A) and in-hospital mortality in (B) Pneumocystis pneumonia (PcP) at Hannover Medical School from 2000 to 2017. Figure S2. Receiver operating characteristic (ROC) curves for in-hospital mortality applied to the total sample. Figure S3. ROC curves for in-hospital mortality applied to subgroups regarding underlying etiology of immunosuppression and for the ICU cohort. Figure S4. ROC curves for in-hospital mortality with the LDH prediction model applied in three strata (years 2000–2005, 2005–2010, 2010–2017). Figure S5. Association between trimethoprim-sulfamethoxazole (TMP-SMX) dose and mortality (DOCX 21 kb) [file 13054_2018_2221_MOESM1_ESM.docx]

**Additional file 1**

**Figure S1** Cumulative incidence **(A)** and in-hospital mortality **(B)** of *pneumocystis* pneumonia (PcP) at Hannover Medical School from 2000-2017

**
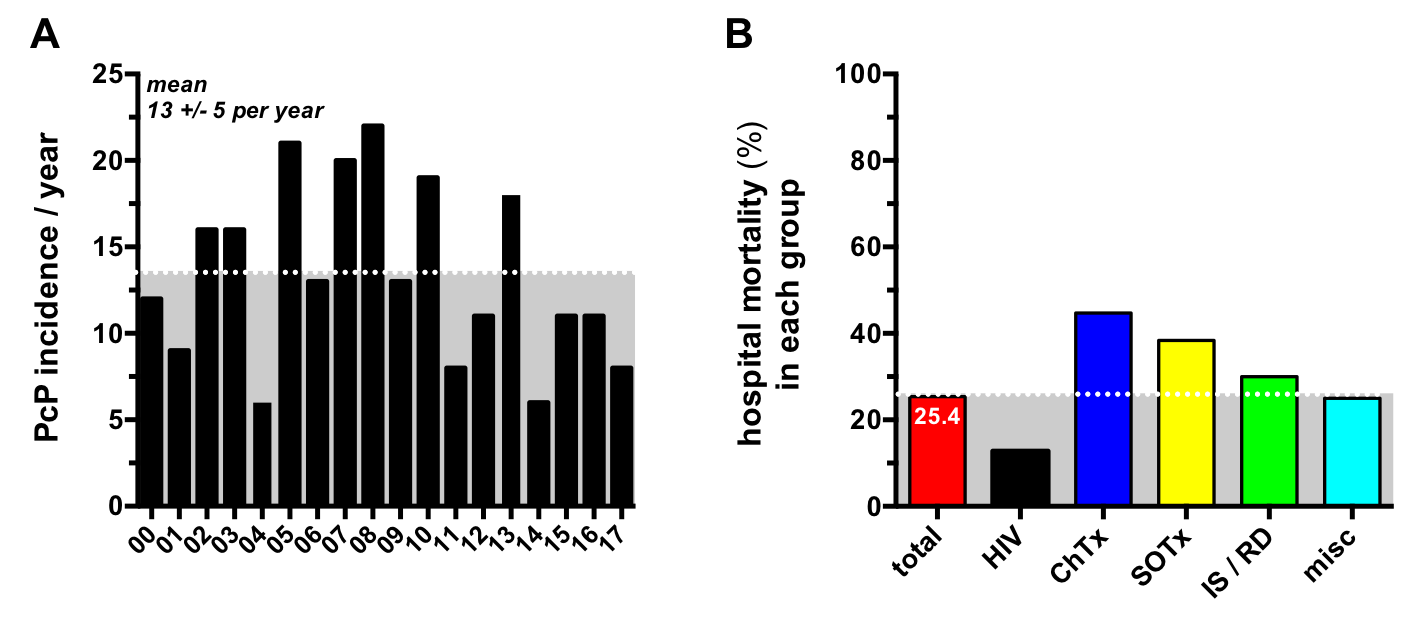
**

**
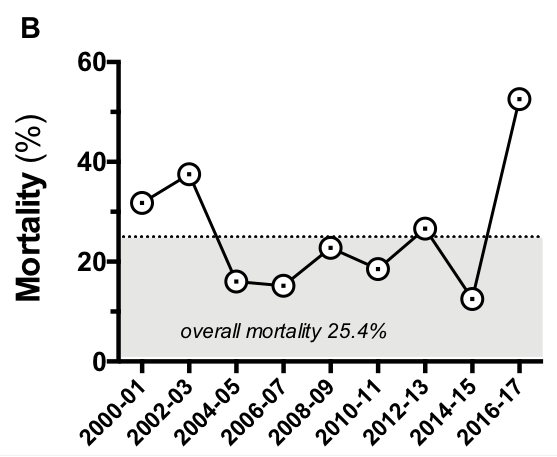
**

**
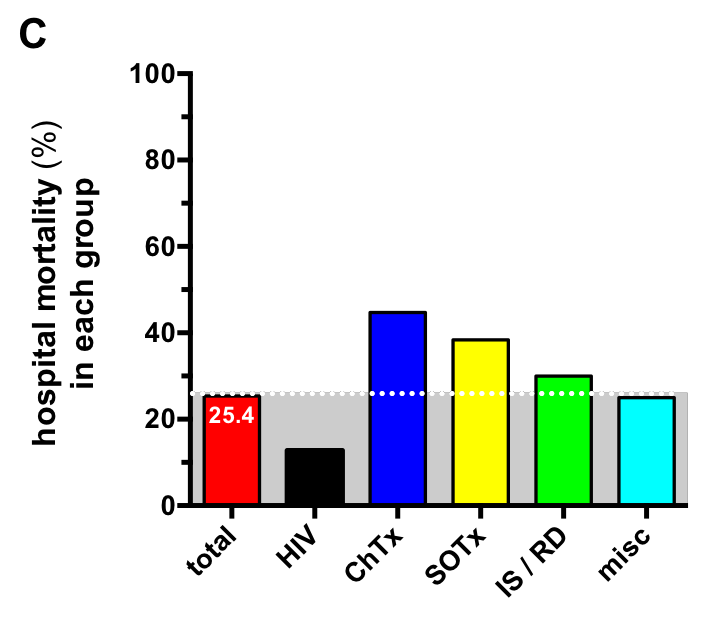
**

Graphs showing **(A)** the annual cumulative incidence of *pneumocystis* pneumonia cases per year from 2000-2017 in our tertiary care center in Germany (grey area highlights the mean PcP cases per year), **(B)** the in-hospital mortality in 2-year intervals, and **(C)** percentage of PcP hospital mortality grouped in etiological subgroups of immunosuppression (HIV – human immunodeficiency virus, ChTx – chemotherapy, SOTx – solid organ transplantation, IS/RD – immunosuppression / rheumatic diseases).

**Figure S2** Receiver operating characteristic (ROC) curves for in-hospital mortality applied to the total sample.

**
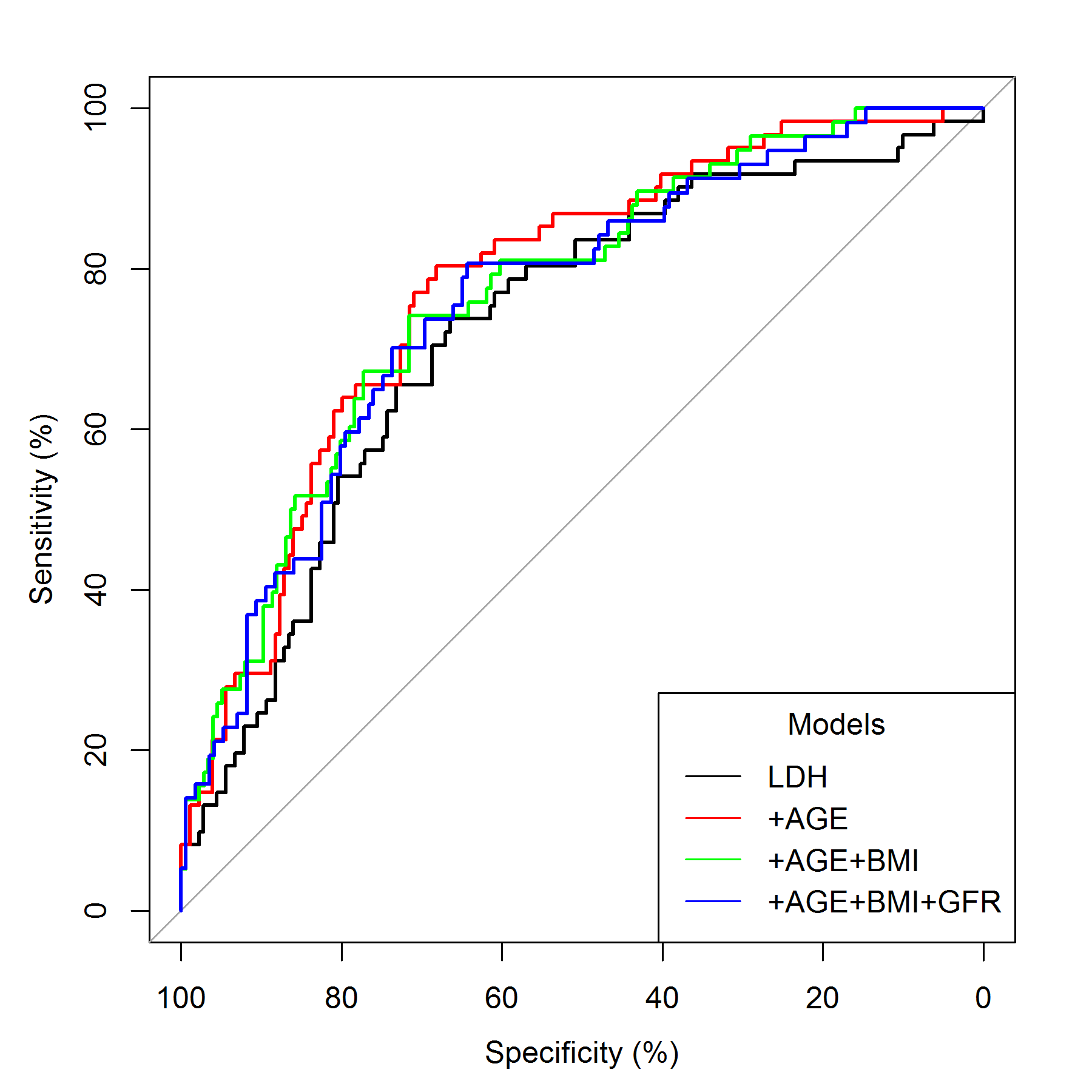
**

Receiver operating characteristic (ROC) curves for LDH (black), LDH + age (red curve) (p=0.06), LDH + age + BMI (green curve) (p=0.18), and for LDH + age + BMI + eGFR (blue curve) (p=0.32). P-values refer to area under the curve (AUC) comparisons to LDH alone (see Methods for details).

**Figure S3** Receiver operating characteristic (ROC) curves for in-hospital mortality applied to subgroups regarding underlying etiology of immunosuppression and for the ICU cohort.

**A) HIV B) CHTx**

**
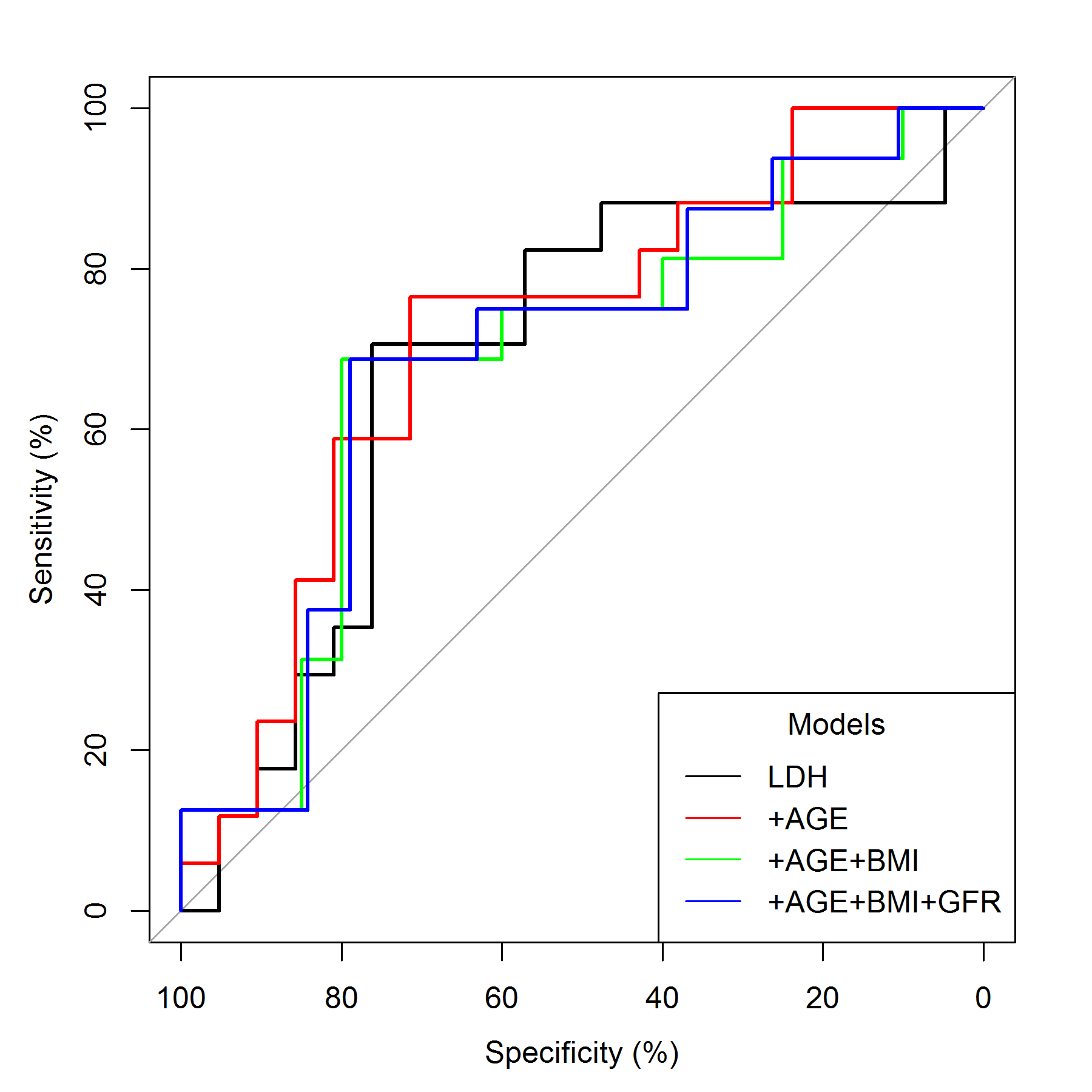

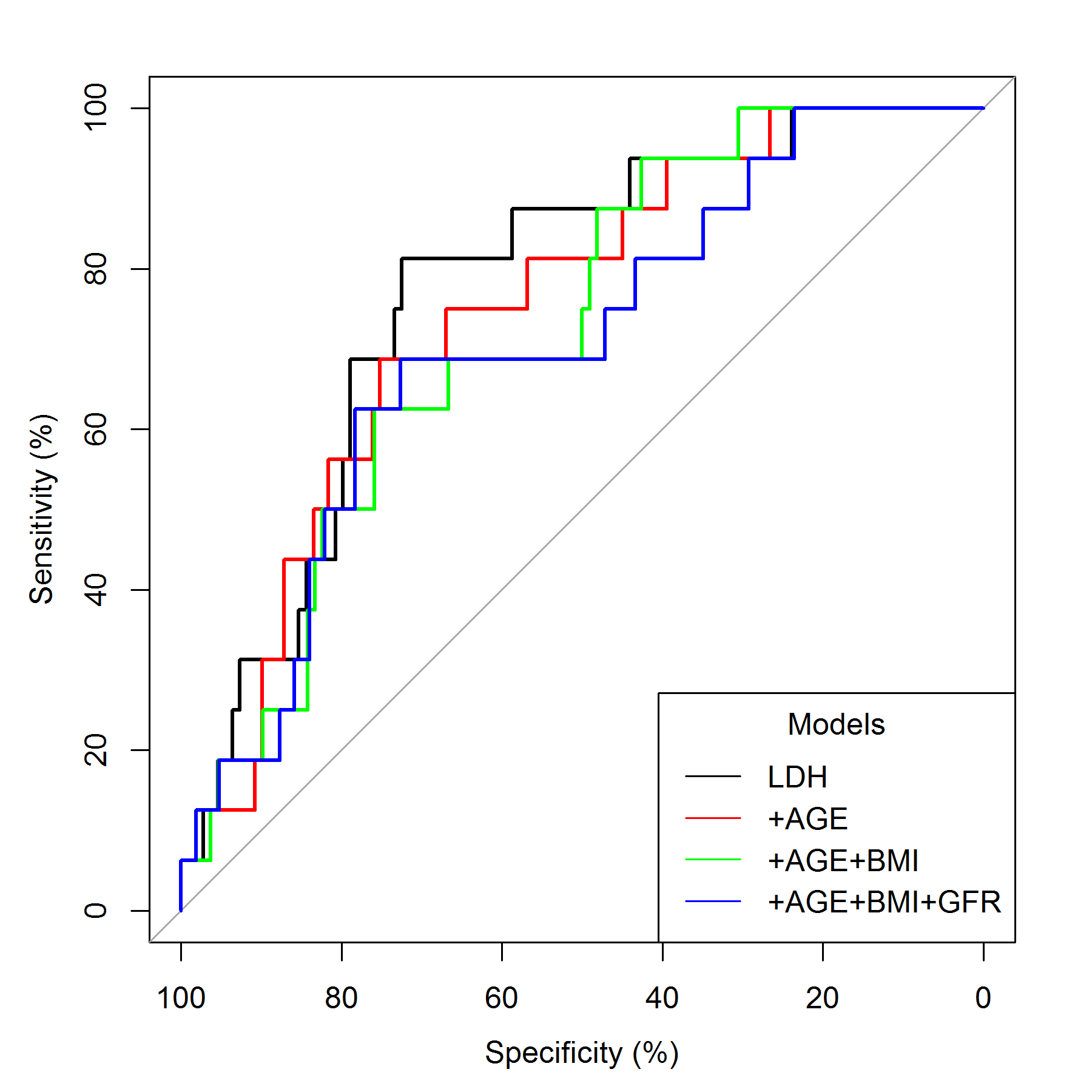
**

**C) SOT D) IS - RD**

**
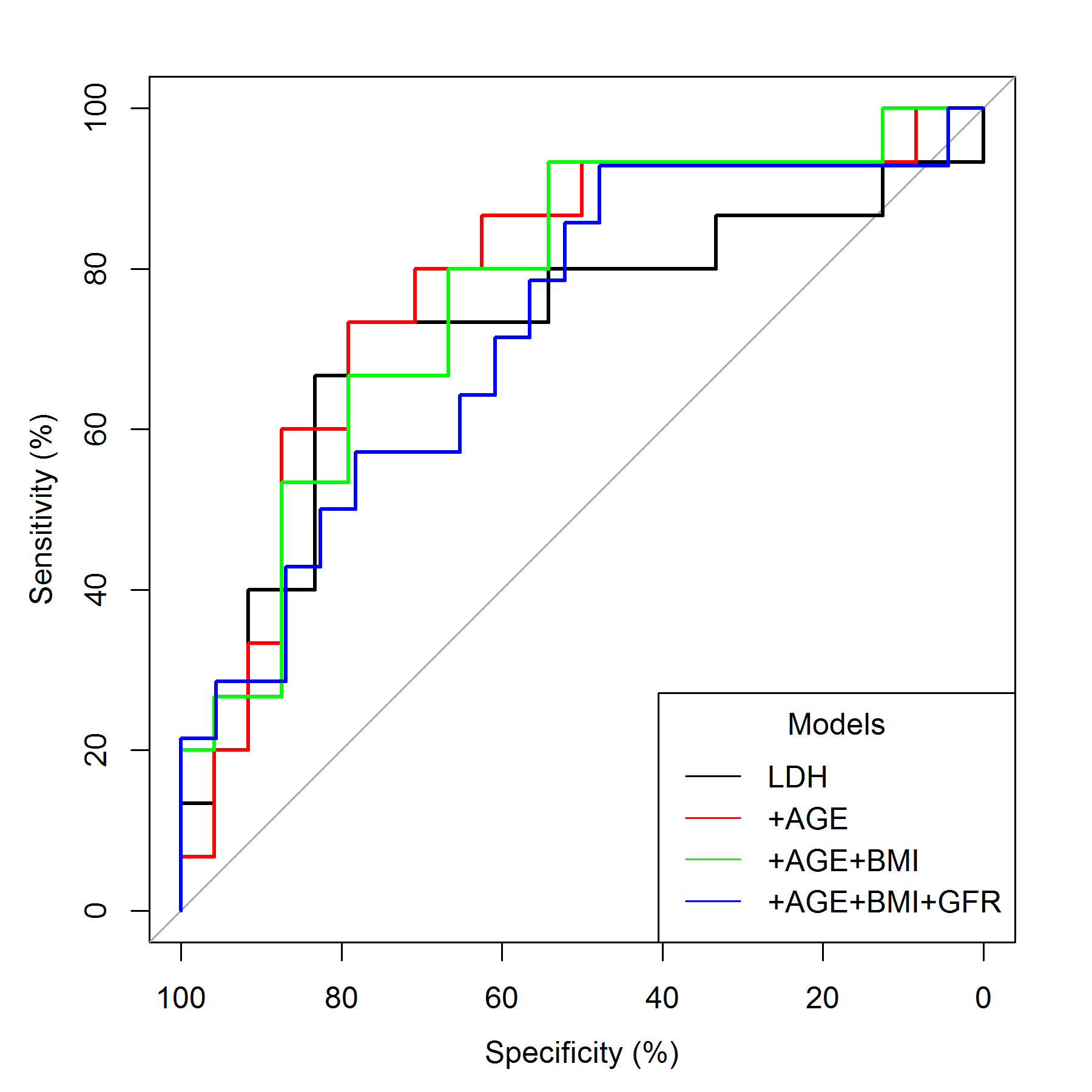

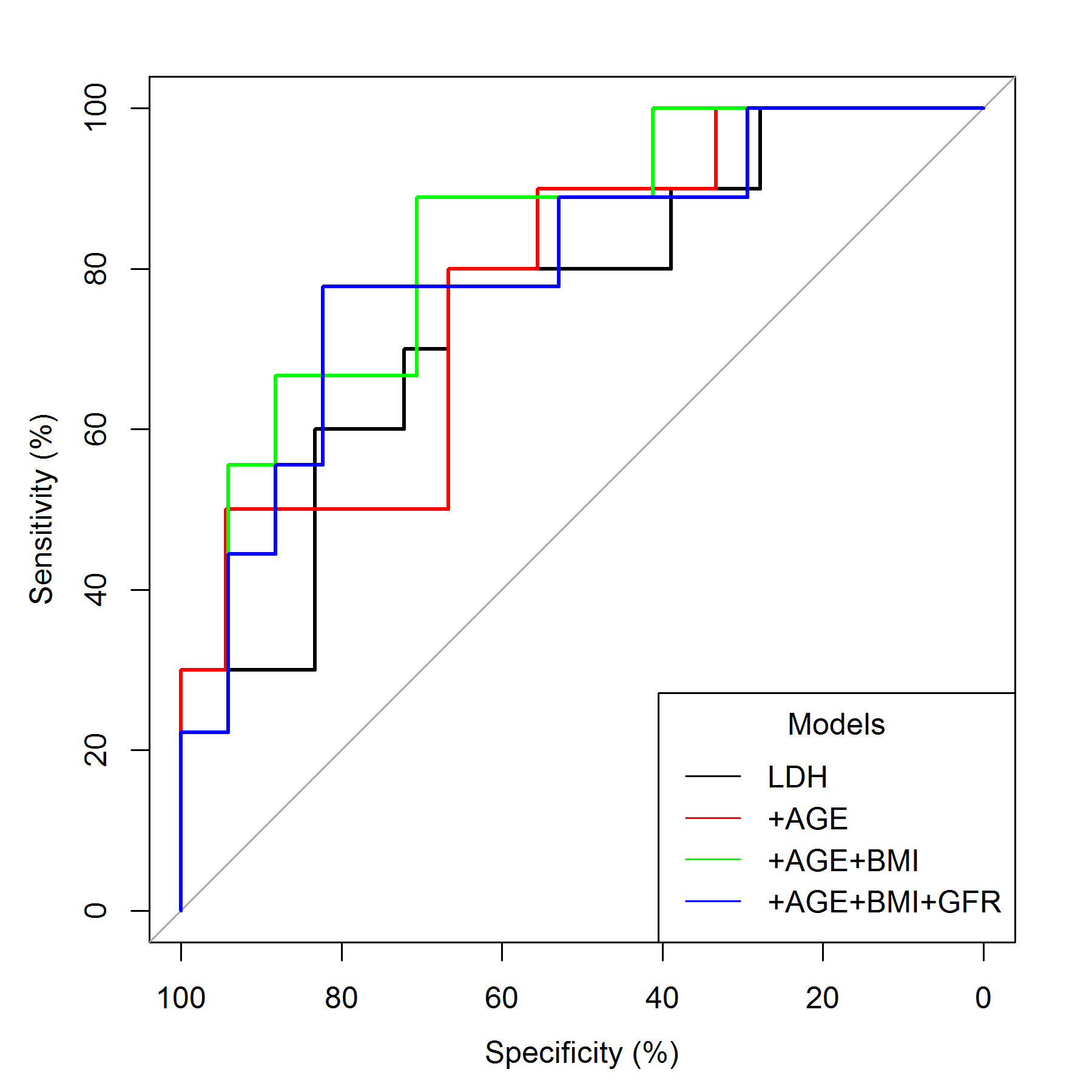
**

**E) ICU patients**

**
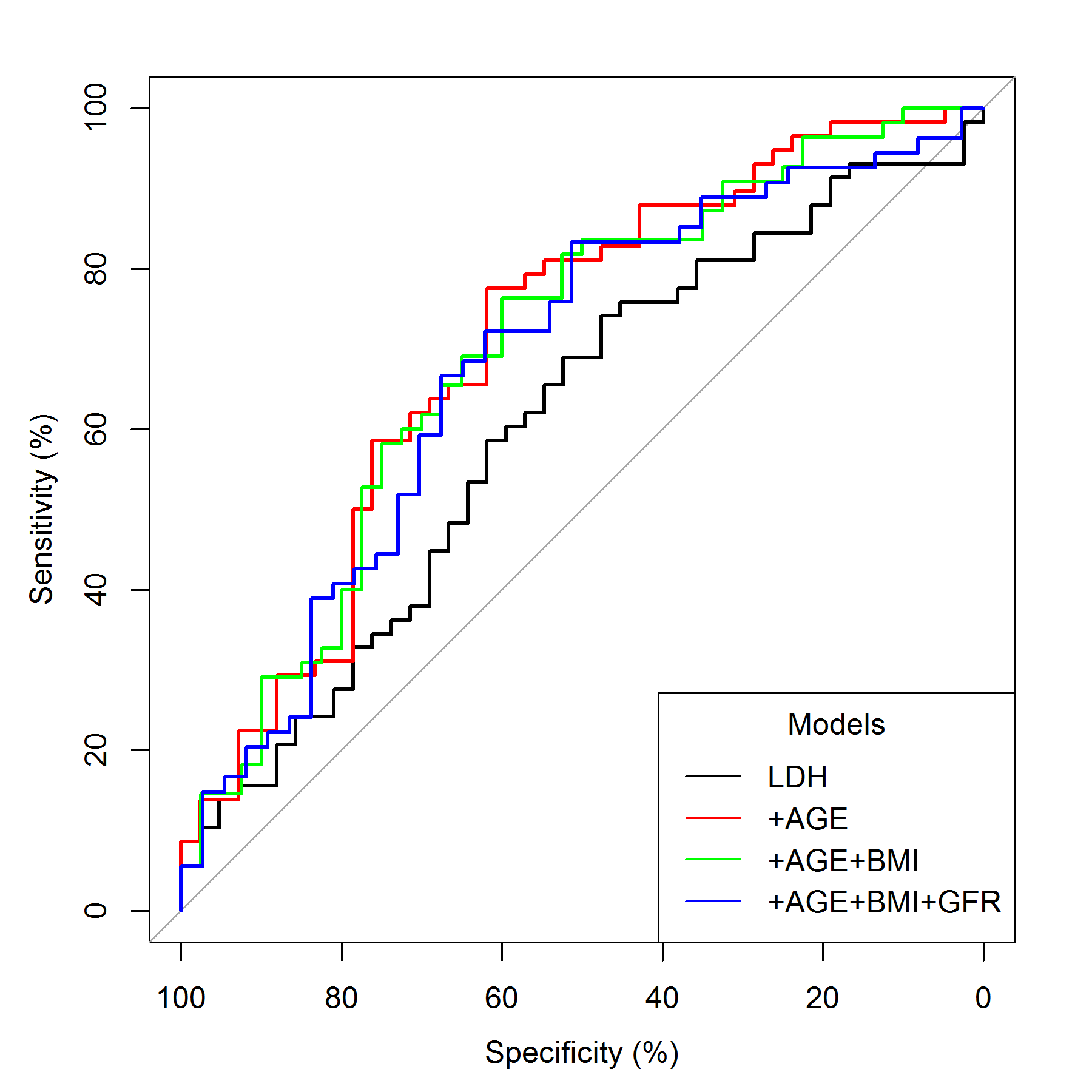
**

**Figure S4** Receiver operating characteristic (ROC) curves for in-hospital mortality applied the LDH prediction model in three strata (years 2000-2005, 2005-2010, 2010-2017)

**A 2000-2005 B 2005-2010**

**
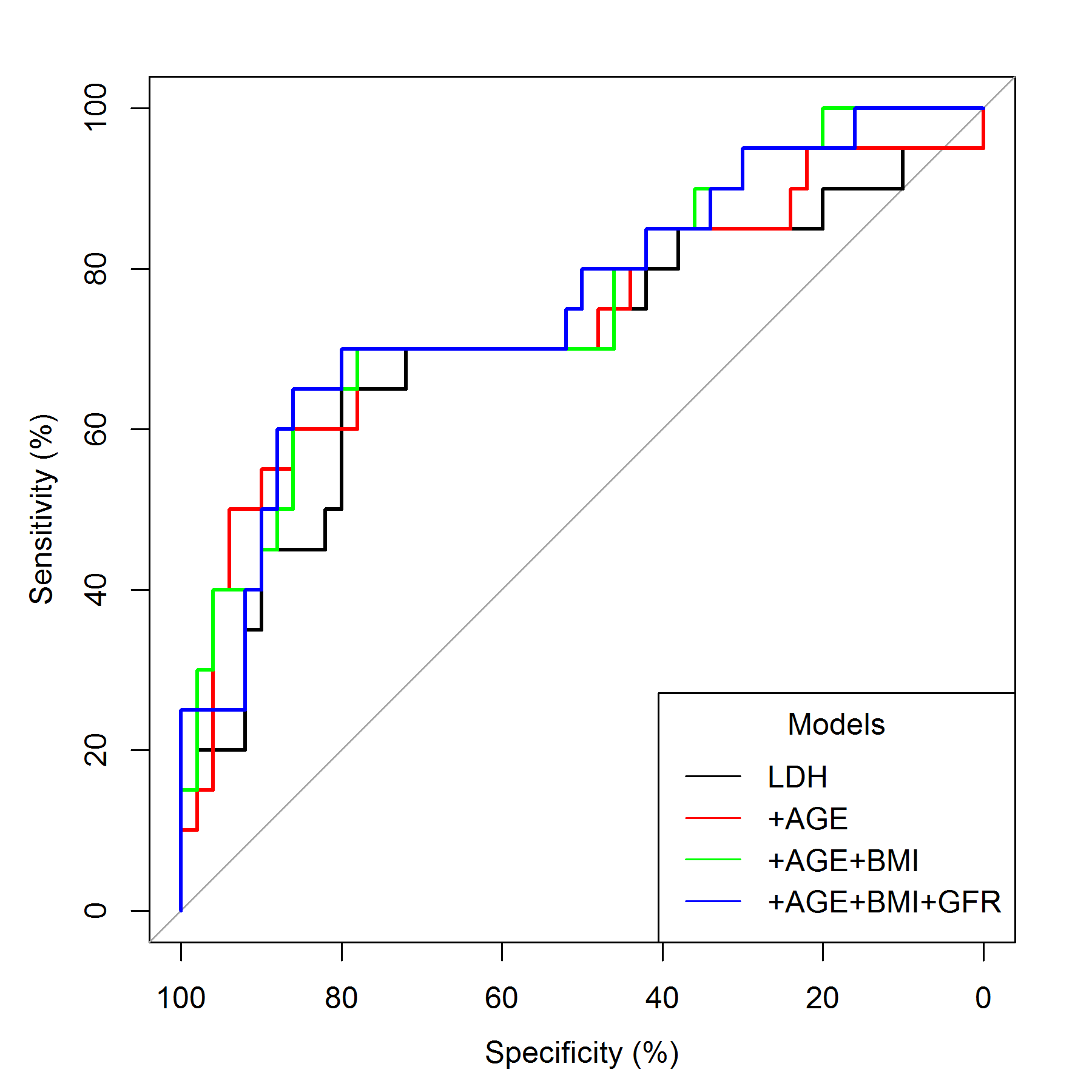

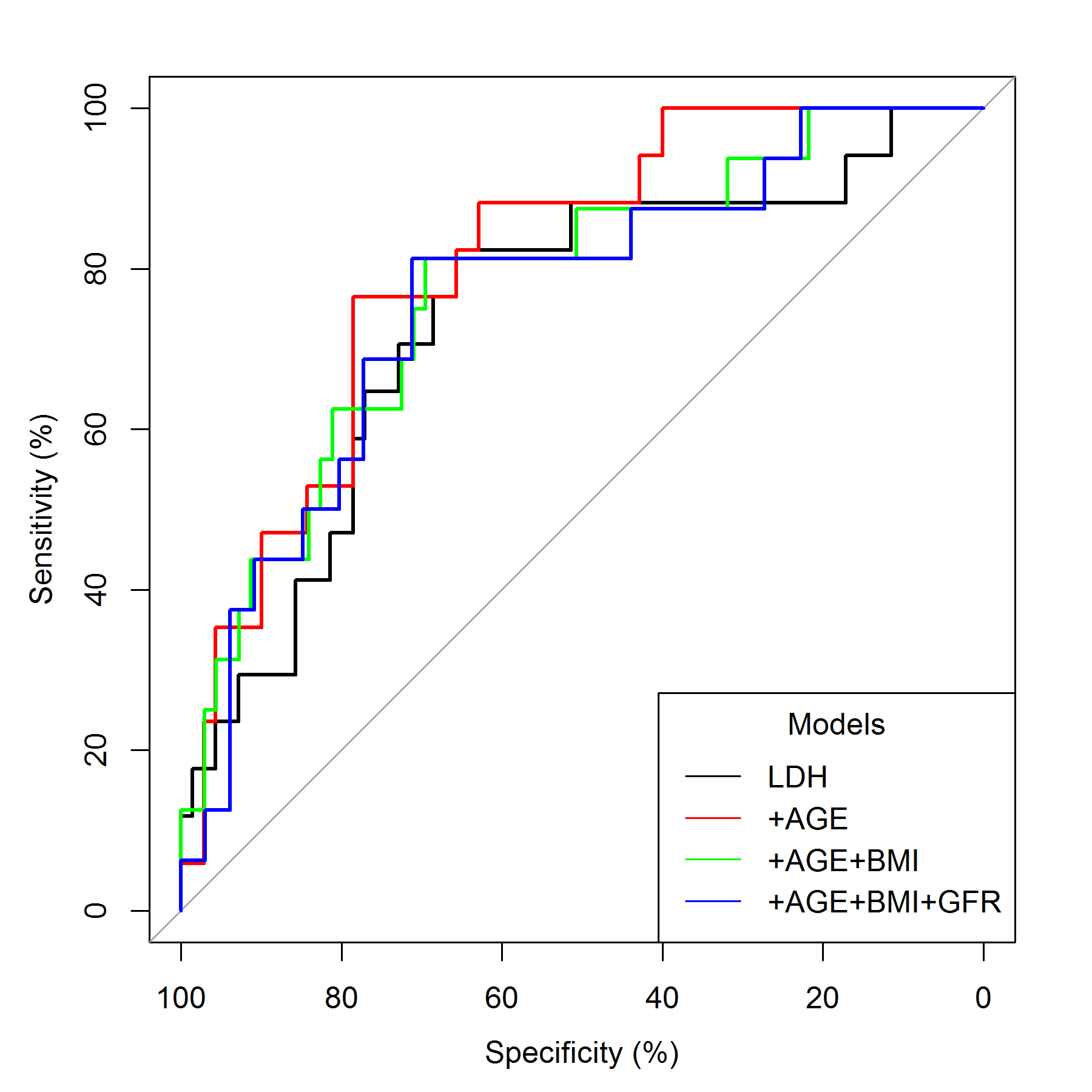
**

**C 2010-2017**

**
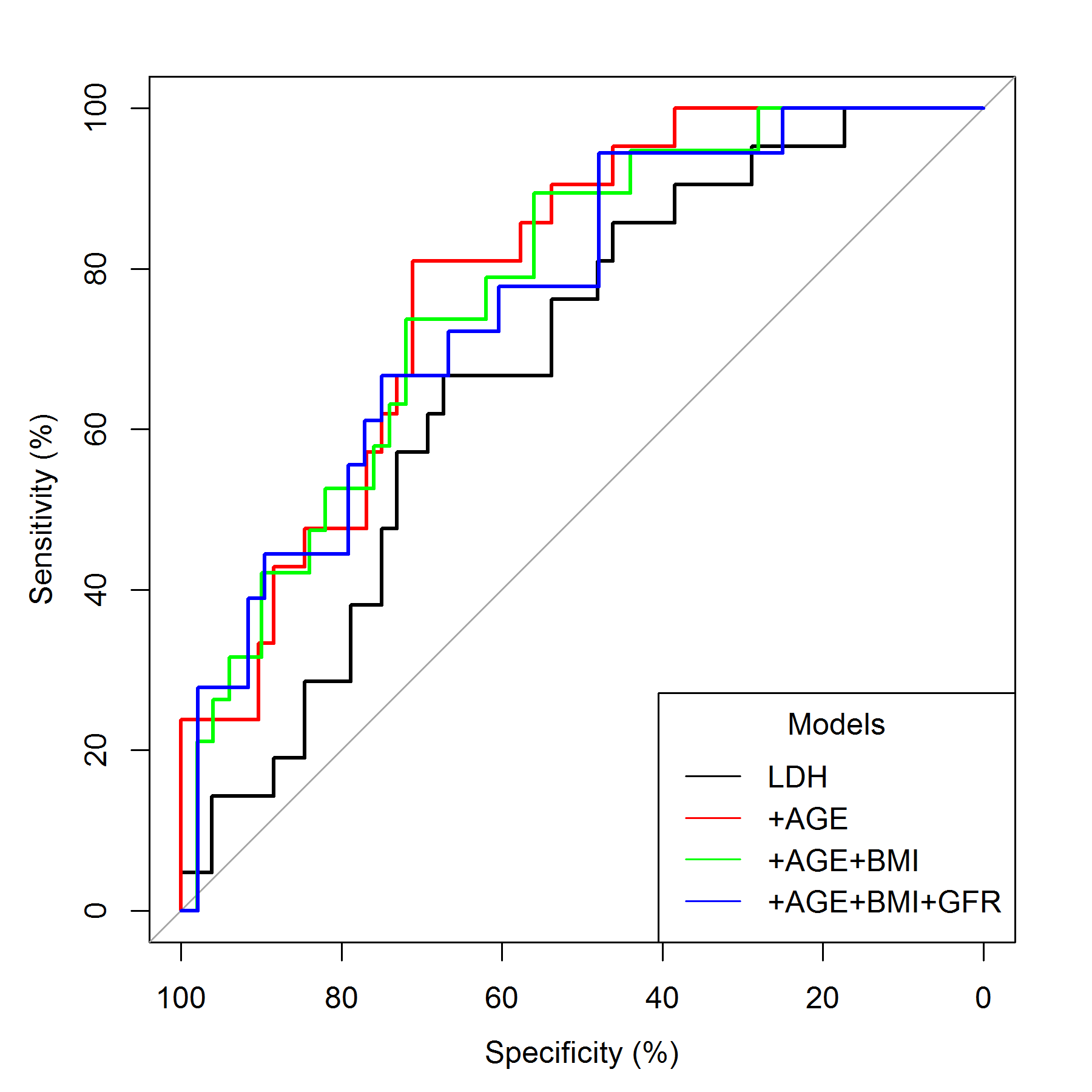
**

**Figure S5** Association of trimethoprim-sulfamethoxazole (TMP-SMX) dose with mortality


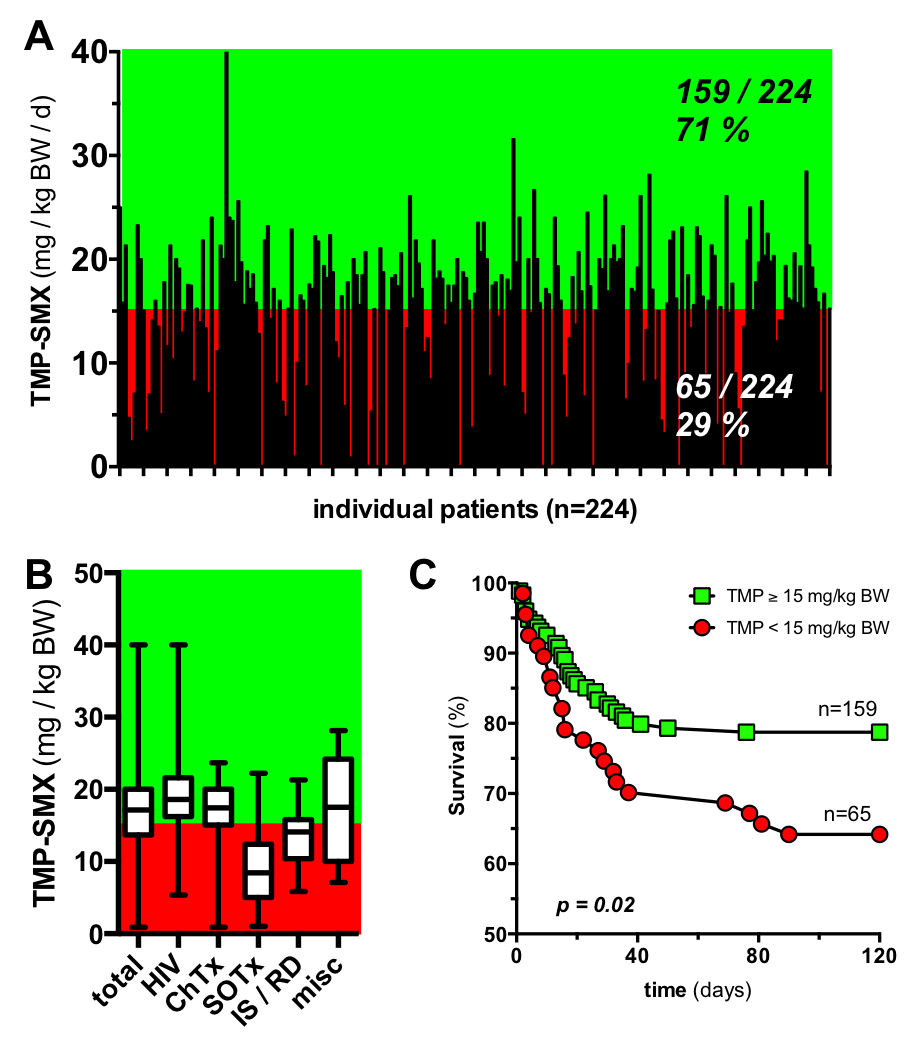


**(A)** Bar graphs show individual doses of trimethoprim-sulfamethoxazole (TMP-SMX) in all 224 patients treated with this standard regimen. Green area highlights the recommended dose ≥ 15 mg/kg bodyweight (BW) (71%) and the red area the dose below < 15 mg/kg (29%). **(B)** Box plots show range of TMP-SMX dose upon different etiologies (HIV – human immunodeficiency virus, CHEMO – chemotherapy, SOT – solid organ transplantation, IS /RD – immunosuppression / rheumatic disease). **(C)** Kaplan-Meier plot shows survival in patients stratified for TMP-SMX dose < and ≥ 15 mg/kg over 120 days (p_Log-rank test_ =0.03).
