# Supplementary material for: Clinical course, treatment and outcome of Pneumocystis pneumonia in immunocompromised adults: a retrospective analysis over 17 years
Source: Crit Care. 2018 Nov 19;22:307. doi: 10.1186/s13054-018-2221-8 (PMC6245758; doi:10.1186/s13054-018-2221-8)
Supplement: Supplementary file 2 — Table S1. Suggested, alternative data-derived LDH cutoff values and their impact on usually reported statistics (with 95% confidence interval in parenthesis) related to the prediction of in-hospital mortality in patients with PcP. Table S2. Estimated AUCs (with 95% confidence interval) of the LDH-related logistic regression models for the prediction of in-hospital mortality in patients with PcP. Table S3. Additional descriptive information (with 95% confidence interval in parenthesis) on patient characteristics and their potential value as predictors of in-hospital mortality in patients with PcP. Table S4. Estimated AUCs (with 95% confidence interval) of the LDH-related logistic regression models for the prediction of in-hospital mortality in patients with PcP. (DOCX 10367 kb) [file 13054_2018_2221_MOESM2_ESM.docx]

**Additional File 2 – Supplemental Tables**

**Table S1:** Suggested, alternative data-derived LDH cut-off values and their impact on usually reported statistics (with 95% confidence interval in parenthesis) related to the prediction of in-hospital mortality in PcP patients.

| LDH  cut-off value^a^ | sensitivity | specificity | positive predictive value | negative predictive value | positive likelihood ratio | negative likelihood ratio |
| --- | --- | --- | --- | --- | --- | --- |
| < 353 U/L | 0.90  (0.80, 0.96) | 0.42  (0.35, 0.50) | 0.35  (0.27, 0.43) | 0.93  (0.85, 0.97) | 1.57  (1.35, 1.82) | 0.23  (0.11, 0.50) |
| < 496 U/L | 0.70  (0.57, 0.81) | 0.70  (0.63, 0.77) | 0.45  (0.35, 0.55) | 0.88  (0.81, 0.92) | 2.38  (1.80, 3.14) | 0.42  (0.28, 0.62) |
| < 755 U/L | 0.25  (0.14, 0.37) | 0.90  (0.85, 0.94) | 0.45  (0.28, 0.64) | 0.78  (0.71, 0.83) | 2.45  (1.31, 4.55) | 0.84  (0.72, 0.98) |

^a^ patients below the cut-off values are regarded are regarded as “test positives”

**Table S2:** Estimated AUCs (with 95% confidence interval) of the LDH-related logistic regression models for the prediction of in-hospital mortality in PcP patients.

| Model | AUC | 95% CI |
| --- | --- | --- |
| LDH | 0.725 | 0.651-0.800 |
| LDH+AGE | 0.777 | 0.711-0.843 |
| LDH+AGE+BMI | 0.767 | 0.700-0.836 |
| LDH+AGE+BMI+GFR | 0.759 | 0.687-0.830 |

**Table S3:** Additional descriptive information (with 95% confidence interval in parenthesis) regarding patient characteristics and their potential value as predictors of in-hospital mortality in PcP patients.

| patient characteristic (potential predictors)^a^ |  | AUC |  | (artificially) dichotomized predictors^d^ | | | | | |
| --- | --- | --- | --- | --- | --- | --- | --- | --- | --- |
|  |  |  |  | sensitivity | specificity | positive predictive value | negative predictive value | positive likelihood ratio | negative likelihood ratio |
| LDH [50 U/L] |  | 0.72  (0.65, 0.80) |  | 0.80  (0.68, 0.89) | 0.60  (0.53, 0.68) | 0.41  (0.32, 0.50) | 0.90  (0.83, 0.95) | 2.03  (1.63, 2.52) | 0.33  (0.19, 0.55) |
| Age  [5 years] |  | 0.67  (0.59, 0.76) |  | 0.62  (0.49, 0.74) | 0.55  (0.47, 0.62) | 0.32  (0.24, 0.41) | 0.81  (0.73, 0.88) | 1.38  (1.07, 1.77) | 0.69  (0.49, 0.98) |
| Sex [REF male]^b^ |  | -- |  | 0.31  (0.20, 0.44) | 0.73  (0.66, 0.80) | 0.28  (0.18, 0.41) | 0.76  (0.69, 0.82) | 1.16  (0.74, 1.81) | 0.94  (0.78, 1.14) |
| BMI [kg/m^2^] |  | 0.64  (0.54, 0.73) |  | 0.66  (0.52, 0.78) | 0.56  (0.48, 0.63) | 0.33  (0.24, 0.42) | 0.83  (0.75, 0.89) | 1.48  (1.15, 1.90) | 0.62  (0.42, 0.90) |
| GFR  [10 mL/min /1.73 m²]^c^ |  | 0.60  (0.51, 0.70) |  | 0.70  (0.57, 0.81) | 0.55  (0.48, 0.63) | 0.35  (0.27, 0.45) | 0.84  (0.76, 0.90) | 1.57  (1.24, 1.99) | 0.54  (0.36, 0.81) |
| prednisone equivalent dose [50 mg/d] |  | 0.63  (0.55, 0.71) |  | 0.63  (0.50, 0.75) | 0.56  (0.49, 0.64) | 0.33  (0.24, 0.42) | 0.82  (0.74, 0.88) | 1.45  (1.13, 1.87) | 0.65  (0.45, 0.93) |
| TMP  [10 kg/d]^c^ |  | 0.51  (0.42, 0.61) |  | 0.58  (0.44, 0.70) | 0.52  (0.44, 0.59) | 0.30  (0.22, 0.39) | 0.77  (0.68, 0.85) | 1.19  (0.91, 1.56) | 0.82  (0.59, 1.15) |

a unit for which effect was estimated or reference (REF) in parenthesis
b females are regarded as “test positives”
c in the dichotomized version lower values were regarded as “test positives”
d note that artificial dichotomization is not recommended (Royston P, Altman DG, Sauerbrei W. Dichotomizing continuous predictors in multiple regression: a bad idea. Stat Med. 2006;25(1):127-41.)

**Table S4:** Estimated AUCs (with 95% confidence interval) of the LDH-related logistic regression models for the prediction of in-hospital mortality in PcP patients.

| Model | AUC | 95% CI |
| --- | --- | --- |
| LDH | 72.46% | 65.07%-79.85% |
| LDH+AGE | 77.71% | 71.14%-84.27% |
| LDH+AGE+BMI | 76.68% | 69.77%-83.58% |
| LDH+AGE+BMI+GFR | 75.85% | 68.71%-82.99% |
